# Non-invasive detection of animal nerve impulses with an atomic magnetometer operating near quantum limited sensitivity

**DOI:** 10.1038/srep29638

**Published:** 2016-07-15

**Authors:** Kasper Jensen, Rima Budvytyte, Rodrigo A. Thomas, Tian Wang, Annette M. Fuchs, Mikhail V. Balabas, Georgios Vasilakis, Lars D. Mosgaard, Hans C. Stærkind, Jörg H. Müller, Thomas Heimburg, Søren-Peter Olesen, Eugene S. Polzik

**Affiliations:** 1 Niels Bohr Institute, University of Copenhagen, Blegdamsvej 17, 2100 Copenhagen, Denmark; 2 Department of Biomedical Sciences, Faculty of Health and Medical Sciences, University of Copenhagen, Blegdamsvej 3, 2200 Copenhagen N, Denmark; 3 Department of Physics, St Petersburg State University, Universitetskii pr. 28, 198504 Staryi Peterhof, Russia

## Abstract

Magnetic fields generated by human and animal organs, such as the heart, brain and nervous system carry information useful for biological and medical purposes. These magnetic fields are most commonly detected using cryogenically-cooled superconducting magnetometers. Here we present the first detection of action potentials from an animal nerve using an optical atomic magnetometer. Using an optimal design we are able to achieve the sensitivity dominated by the quantum shot noise of light and quantum projection noise of atomic spins. Such sensitivity allows us to measure the nerve impulse with a miniature room-temperature sensor which is a critical advantage for biomedical applications. Positioning the sensor at a distance of a few millimeters from the nerve, corresponding to the distance between the skin and nerves in biological studies, we detect the magnetic field generated by an action potential of a frog sciatic nerve. From the magnetic field measurements we determine the activity of the nerve and the temporal shape of the nerve impulse. This work opens new ways towards implementing optical magnetometers as practical devices for medical diagnostics.

The magnetic field generated around a signaling nerve fiber is of key interest both from a basic scientific and a clinical point of view. The transmembrane potentials have been extensively measured with electrophysiological techniques. Magnetic field measurements are insensitive to the transmembrane currents as the fields from the opposite currents in and out of the membrane cancel. Instead, magnetic field measurements allow for a true measurement of the axon’s axial net current, which is the depolarizing wavefront driving the action potential. Magnetic field recordings also allow for non-invasive measurements of the conduction velocity of peripheral nerves^1^ which is necessary for diagnostics of multiple sclerosis, myotonia and intoxication in patients.

The magnetic field of a nerve impulse was first measured by Wikswo *et al*.^2^ using a combination of a superconducting SQUID magnetometer and a toroidal pick-up coil through which the nerve had to be pulled. This method is not compatible with *in vivo* diagnostics and yields the magnetic field values which are much higher than that in an animal because the return currents in the surrounding tissue are not measured. Here we are able to detect the nerve impulse with the sensor placed beside the nerve, several millimeters away, the setting compatible with *in vivo* studies. Detection of nerve impulses with a magnetometer based on Nitrogen-Vacancy centers in diamond has recently been reported^3^. Such magnetometers seem promising for magnetic field microscopy applications where the magnetometer can be placed at micrometer distance or closer to the biological object.

Sensitivity of atomic magnetometers^4^ improves with the number of atoms sensing the field, which for vapor magnetometers is defined by volume and temperature. For example, femtoTesla sensitivity has been achieved with magnetometers operating at a temperature of several hundred of °C in the so-called SERF regime^5^ used also for medical applications^6^,^7^,^8^. Similarly high sensitivity has been achieved at room temperature using much fewer atoms by means of quantum state engineering^9^ leading to operation beyond standard quantum limits of sensitivity. Room temperature operation allows to place the sensor in contact with the skin or potentially inside the human body. The close proximity of the sensor to the source of magnetic field is a big advantage as the magnetic field rapidly decreases with the distance from the source. Room-temperature cesium magnetometer has been used for medical applications^10^, however, it operated far above quantum limits of sensitivity.

## Results

**Figure 1:**
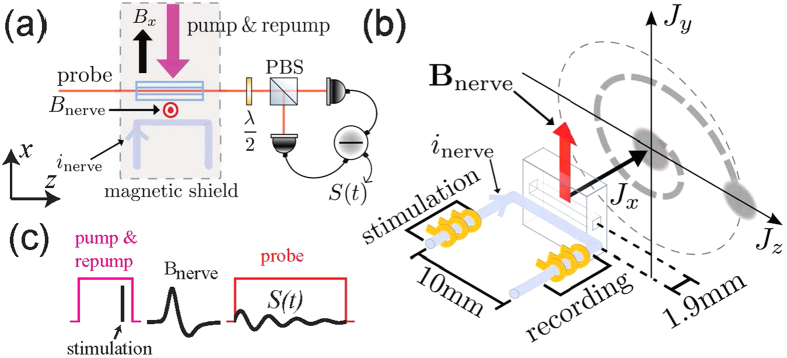
(**a**) Schematic of the experimental setup. Probe light propagates along the *z* axis. Half-wave plate *λ*/2, polarizing beam splitter (PBS) and differential photodetection are components of polarization detector. (**b**) The magnetometer principle. The amplitude of the collective atomic spin precession in *z*, *y* plane is proportional to *B*_nerve_. Spin projection *J*_*z*_ is measured by probe light with the sensitivity limited by the quantum projection spin noise (fuzzy circle). The magnetic field from the nerve is circumferal. The average field detected by the magnetometer points in the *y*-direction. (**c**) The measurement sequence for the pulsed magnetometer mode.

Here we use the approach of ref. 9 for nerve impulse measurements. The sensitive element of the magnetometer is cesium atomic vapour. Cesium has a high vapor pressure such that high sensitivity can be reached at room- or human body temperature. The magnetic moment (spin) of atoms **J** = (*J*_*x*_, *J*_*y*_, *J*_*z*_) is prepared by optical pumping in the *x*-direction, along the direction of a bias field *B*_*x*_ [see Fig. 1(a)]. The magnetic field of the nerve *B*_nerve_ will create a transverse spin component **J**_⊥_ = (*J*_*y*_, *J*_*z*_) which afterwards will rotate in the *y*-*z* plane at the Larmor frequency Ω = *B*_*x*_/*γ* [see Fig. 1(b)], where *γ* = 2.20 · 10^10^ rad/(s · T) is the cesium gyromagnetic ratio. The *J*_*z*_ spin component is detected optically by measuring the polarization rotation of the probe light. The magnetic field from the nerve is detected in two modalities, a continuous mode where the magnetic field as a function of time *B*(*t*) is detected, and a pulsed mode where the Fourier component |*B*(Ω)| is detected. In the continuous mode the pump and probe light is continuously on. In the pulsed mode [see Fig. 1(c)], a pulse of pump light is followed by the pulse of magnetic field, and finally the spins are detected with a pulse of probe light.

Optical magnetometers are fundamentally limited by quantum noise consisting of the spin-projection noise (PN) shown as the fuzzy circle in Fig. 1(b), quantum shot noise of the probe light and quantum back action of the probe on the spin^9^. For an optimal measurement these noise sources add up to the standard quantum limit (SQL)^11^. This limit has been reached for magnetic fields oscillating at hundreds of kHz^9^. Here we approach the SQL for the magnetic field measurement of nerve impulses whose frequency is much lower using the techniques described in the Supplementary Information. For continuous measurements, the SQL magnetic field uncertainty Δ*B*_SQL_ normalized by the total measurement time *T*_tot_ yields the sensitivity 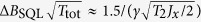 in units of 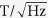. *T*_2_ is the spin coherence time and *J*_*x*_ = 4*N*_*A*_ is the total atomic spin for *N*_*A*_ cesium atoms. At room temperature of 22 °C, the cesium atomic density is 3.1 × 10^16^ m^−3^ which is the highest of all elements appropriate for atomic magnetometry. The pulsed measurement has the SQL magnetic field uncertainty of 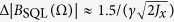 if the magnetic pulse duration *τ* ≪ *T*_2_ (see Supplementary Information).

A long spin-coherence time *T*_2_ is crucial for a high sensitivity. In this work we utilize a vapor cell with the inside surface coated with alkane^12^,^13^. The coating protects atomic spin states from decoherence over many thousands of wall collisions and provides 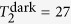 ms which is longer than a typical nerve impulse duration *τ* ≈ 2 ms, as required for the ultimate sensitivity. The cesium vapor is contained in a 1.0 mm × 1.0 mm × 7.7 mm channel inside a rectangular glass chip of dimensions 2.3 mm × 8.4 mm × 7.7 mm. This small rectangular vapor cell allows us to have atoms at an average distance of a few mm from the nerve, which is close to a typical distance for many medical applications.

**Figure 2:**
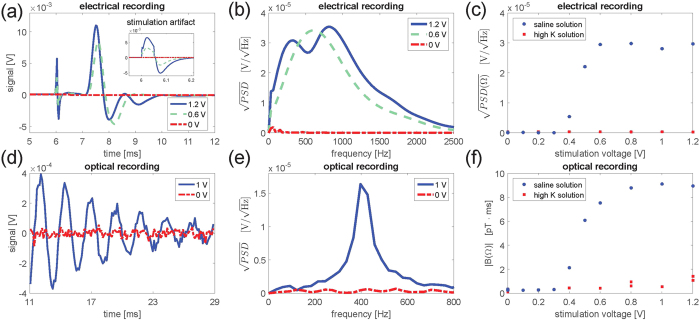
Electrical and optical measurements of the nerve impulse for different stimulation voltages. The optical measurements were done in the pulsed mode using 1000 averages. The figures show the signals in time, the square-root of the power spectral density *PSD* and the 400 Hz frequency component. The plotted electrical signals are after 10 times amplification. The uncertainties on the data points in (**c**) are to small to be visible in the figure. The uncertainties on the points in (**f**) can be estimated from the points without stimulation (0 V) which were measured 9 times and resulted in a 0.25(10) pT·ms signal. By dividing the nerve signal (9.1 pT·ms) by the noise floor obtained without stimulation (0.25 pT·ms) we find the signal to noise ratio, *SNR* ≈ 37.

A frog sciatic nerve contains a few nerve bundles each with several thousand axons inside (see Methods section). The nerve is placed inside a plastic chamber where it can be kept alive in a saline solution for more than 5 hours. The nerve is electrically stimulated from one end with a pair of gold electrodes [see Fig. 1(b)]. The stimulus triggers an action potential (a nerve impulse) propagating along the nerve. As a reference measurement we perform an electrical recording of the impulse with another pair of electrodes. Figure 2(a) shows the electrically recorded signals for different stimulation voltages. Figure 2(b) shows the frequency spectra of the nerve signals and Fig. 2(c) shows the amplitude of the 400 Hz Fourier component. The nerve is stimulated at *t* = 6.0 ms. The signature of the nerve signal is its non-linear behavior with stimulation voltage, with a firing threshold at around 0.4 V. For voltages above the threshold a nerve impulse is measured with the recording electrodes within the time interval *t* = 7.0 − 10.0 ms. We also observe a stimulation artifact at *t* = 6.0 ms [see inset in Fig. 2(a)] which is proportional to the stimulation voltage.

In parallel to the reference electrical recording the nerve signal is detected optically using the pulsed magnetometer mode. The magnetometer is positioned near the middle part of the nerve separated only by a thin microscope cover slip. As the nerve is bent in a U-shape, we mainly detect the field from the 10 mm section of the nerve closest to the magnetometer [see Fig. 1(b)]. The axial ionic current in this 10 mm section can be considered constant, as the action potential has a duration ≈3 ms, the velocity ≈30 m/s, and therefore an extent ≈9 cm ≫ 10 mm. The circumferal magnetic field **B**_nerve_ from the nerve is on average transverse to the initial spin direction *J*_*x*_ and will therefore create a transverse spin component [see Fig. 1(b)]. Figure 2(d) shows the magnetometer signal, Fig. 2(e) shows the spectrum and Fig. 2(f) shows the magnetic field Fourier component at the Larmor frequency of 400 Hz. A clear threshold for the nerve firing is observed confirming that the magnetometer is capable of detecting the nerve impulse. From calibration measurements (see Methods section) we determine the Fourier component of the magnetic field from the nerve as |*B*_nerve_(Ω)| = 9.1 pT·ms. From Fig. 2(f) we infer that the nerve impulse can be detected optically with a signal to noise ratio *SNR* ≈ 37 using 1000 averages. As the *SNR* scales as 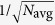 we find the *SNR* ≈ 1.2 for a single shot, i.e., it should be possible to detect a nerve impulse in a single shot.

As a control experiment, we make the nerve inexcitable^14^ by replacing the saline solution in the plastic chamber with a solution with high potassium concentration. As expected, we clearly observe from both electrical [Fig. 2(c)] and optical [Fig. 2(f)] measurements that the nerve signal is blocked by this solution. Note that the stimulation artifact which was observed in the electrical recording (inset to Fig. 2(a)) should be small in the optical measurements, as the stimulation occurs during the optical pumping [see Fig. 1(c)] where the response to magnetic fields is strongly damped. When the nerve is inexitable, we do see such a small stimulation artifact (squares in Fig. 2(f)) for the higher stimulation voltages.

**Figure 3:**
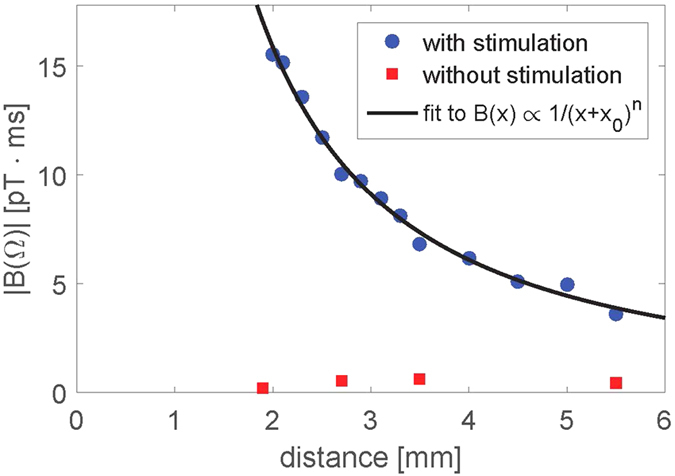
Magnetic field fourier component as a function of distance (using 1000 averages per point). The closest distance from the center of the magnetometer to the center of the nerve was estimated to be 1.9 mm. Circles: Nerve magnetic field, squares: magnetometer noise floor, line: fit to a power-law dependence, fit parameters *n* = 1.5(4), *x*_0_ = 0.2(8) mm. The uncertainties on the fit parameters are the 68% confidence bounds.

The high spatial resolution of our magnetometer (the channel containing the cesium atoms is 1.0 mm across) and the good signal to noise ratio allows us to characterize how the magnetic field decays with distance from the nerve. The results are shown in Fig. 3 and one sees that we can detect the nerve impulse more than 5 mm away. The actual distance dependence may be complicated as the frog sciatic nerve contains 1000’s of axons organized in several bundles (see Supplementary Fig. 1). However, we expect a power law dependence 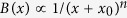 with *n* between 1–3. *n* = 1 corresponds to the magnetic field from an infinitely long wire and *n* = 3 corresponds to the field from a magnetic dipole. *x*_0_ is the position of the nerve relative to our estimate of 1.9(5) mm based on the size of the vapor cell and dimensions of the nerve. Fitting the data to a power law dependence yields *x*_0_ = 0.2(8) mm and *n* = 1.5(4) which are within the expected ranges.

**Figure 4:**
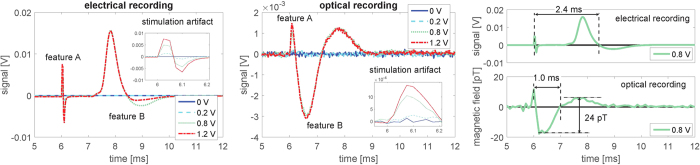
Electrical and optical measurements of the nerve impulse for different stimulation voltages. The magnetometer was operated in the continuous mode and the signals were averaged 5000 times. (**a**) Electrical and (**b**) optical measurements. (**c**) Electrical signal for 0.8 V stimulation and magnetic field calculated by deconvolution. For these specific measurements the Larmor frequency was 510 Hz and the coherence time 0.37 ms.

The magnetometer can also be operated in the continuous mode which allows for determination of the temporal shape of the magnetic field generated by the nerve, *B*_nerve_(*t*). The magnetometer response was optimized by matching its frequency response (a Lorentzian centered at the Larmor frequency with a full width at half maximum 1/(*πT*_2_)) with the spectrum of the nerve impulse (see Fig. 2(b)). The bandwidth 1/(*πT*_2_) = 860 Hz was set by choosing suitable power levels for the pump, repump and probe lasers. Figure 4(a,b) show the electrical and optical signals respectively as a function of time for different stimulation voltages. In both electrical and optical measurements we observe two features (A and B). Feature A is due to the stimulation as it starts at the time of stimulation and increases linearly with the stimulation voltage. Feature B is due to the nerve signal, as it last for several ms and only appears above the threshold for the nerve firing (here 0.8 V or greater). Figure 4(c) shows a comparision of the electrical signal for 0.8 V stimulation and the detected magnetic field *B*(*t*) as calculated by deconvolving the optical signal with the magnetometer response [see Methods]. The temporal profiles of the electrical signal and the magnetic field look very similar; both show the action potential and a stimulation artifact. From the bottom plate of Fig 4(c) we conclude that the nerve magnetic field has a 24 pT peak-to-peak amplitude (measured at an average distance of 1.9 mm) and that the nerve conduction velocity is 34(8) m/s (see Supplementary Information). The effective axial ionic current is estimated to be 0.23 *μ*A (see Supplementary Information) which is consistent with earlier measurements^15^.

From the data we find the single shot experimental uncertainty Δ|*B*_exp_(Ω)| = 7.7 pT·ms for the pulsed mode and a sensitivity of 230 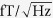 in the continuous mode (see Methods section). The standard quantum limit for the magnetic field uncertainty is in the pulsed mode Δ|***B***_SQL_(Ω)| = 1.6 pT·ms. In this mode the light is off during the nerve impulse duration *τ* ≈ 2 ms which satisfies 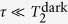. In the continuous mode, where the *T*_2_ = 0.37 ms matches the nerve impulse bandwidth, the SQL for the sensitivity is 160 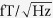. As the experimental sensitivity is close to the SQL, quantum noise (projection noise, shot noise, back-action noise) is a considerable fraction of the total noise. Some uncompensated low frequency classical noise of the probe light and of the atomic spin also contributed to the total noise, in particular for the pulsed measurements.

## Discussion

Projection noise dominated sensitivity can be reached by relatively straightforward steps, such as using multipass vapor cells^16^, by modest heating (increasing the temperature from room-temperature 22 °C to the human body temperature 37 °C will increase the sensitivity by a factor of two^17^) or by employing a low finesse optical cavity^11^. Gradiometry with two cells with oppositely oriented spins allows for generation of nonclassical entangled states leading to sensitivity beyond the PN limit^9^ as well as provides additional compensation of the ambient magnetic fields and classical fluctuations of the atomic spins.

In conclusion, we have performed non-invasive detection of nerve impulses from the frog sciatic nerve by measuring the magnetic field generated by the nerve with a room-temperature sensor with near quantum limited sensitivity. A mm-sized sensor which is sensitive enough to detect sub-picoTesla fields at a distance of a few millimeters from biological objects makes the magnetometer perfect for medical diagnostics. We envision that practical, low-cost, and room-temperature atomic magnetometers could be an alternative to the current technology based on cryogenically-cooled SQUID magnetometers which are widely used for magnetoencephalography (MEG) and neuroscience studies^18^,^19^. Promising applications of atomic magnetometers include MEG^20^, detection of epilepsy^8^, detection of the synaptic responses in the retina, and cardiography of fetuses^6^.

## Methods

### Nerve preparation

This study was conducted in accordance with the University of Copenhagen Animal Ethics Policy^21^ and the frogs used were handled by the animal facility at the Department of Experimental Medicine at the University of Copenhagen which is accredited by the Association for Assessment and Accreditation of Laboratory Animal Care International (AAALAC)^22^. The experiments presented here with the frog sciatic nerve do not require a license.

Sciatic nerves were isolated from green frogs (Rana Esculenta). The frogs were decapitated and the sciatic nerves were isolated from spine and down to the knee. The nerves are 7–8 cm long with a diameter of 1.3 mm in the proximal end and slightly thinner in the distal end. In the proximal end there is one bundle that divides twice so distally it is composed of three bundles. Supplementary Fig. 1 shows an electron micrograph of the nerve where it is seen that the nerve bundles contain a few thousand of axons.

Throughout the dissection and the course of the experiments, the frog sciatic nerves were kept moist in cold Ringers solution (also called saline solution) of 115 mM NaCl, 2.5 mM KCl, 1.8 mM CaCl_2_, 1.08 mM Na_2_HPO_4_ · 2H_2_O, 0.43 mM NaH_2_PO_4_ · H_2_O, adjusted to pH 7.1^23^. Ringers solution approximates the ionic composition of the extracellular fluids of the frog. A high potassium concentration Ringers solution, in which all NaCl was replaced by KCl with final K^+^ concentration being 117mM and Na^+^ 0 mM and Cl^−^ concentration remaining constant, was used to make nerves inexcitable^14^.

### Nerve chamber and electrical recording

The nerves are kept in a 3D-printed plastic chamber during the experiments. The chamber is a 47 × 18 mm block of 8.5 mm height that contains a longitudinal U-shaped channel with a diameter of 2 mm in which the nerve is placed. The channel allows maintenance of a saturated water-vapor atmosphere in order to keep the nerve moist. The front part of the chamber is placed close to magnetometer and it is covered by a microscope glass cover slip of 0.13 mm thickness.

The chamber has 6 circular gold electrodes on each side. The electrodes have an outer diameter of 6 mm with the hole inside of 1.5 mm, which fits into the channel. On each side, the distance between electrodes is 5 mm.

The nerve was externally stimulated in the proximal end by applying a short 50 *μ*s square voltage pulse between two spatially separated electrodes surrounding the nerve. The nerve signal was measured in the distal end as a potential difference between a pair of two electrodes. The electrical signal was amplified 10 times and filtered (using a 3 kHz low pass and a 10 Hz high pass filter) and recorded simultaneously with the magnetic field recordings.

### Operation of the magnetometer

The magnetometer is based on optical read-out of spin-polarized atomic vapour. The cesium atoms are prepared by an optical pumping with a pulse of circular polarized light such that the total spin vector **J** = (*J*_*x*_, *J*_*y*_, *J*_*z*_) points in the *x*-direction, which is also the direction of a bias field *B*_*x*_ [see Fig. 1(a)]. Any magnetic field perpendicular to *x*-direction (such as the magnetic field from the nerve) will create a transverse spin component **J**_⊥_ which afterwards will precess around the bias magnetic field at the Larmor frequency Ω = *B*_*x*_/*γ* [see Fig. 1(b)], where *γ* = 2.20 · 10^10^ rad/(s · T) is the cesium gyromagnetic ratio. The transverse spin component is detected optically by measuring the polarization rotation of linearly polarized probe light passing through the vapor cell using a balanced polarimeter. The magnetic field from a nerve can be detected in two modalities, a pulsed or a continuous one. In the continuous mode the pump and probe light is continuously on. In this case, the magnetometer signal *S*(*t*) is proportional to the convolution of the magnetic field with the magnetometer response function 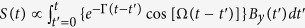, where we assumed the transverse field is along the *y*-direction. The relaxation rate Γ = 1/*T*_2_, which is the inverse of the spin-coherence time *T*_2_, increases linearly with laser power and is in the limit of low power denoted 
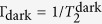
. In the pulsed mode [see Fig. 1(c)], a pulse of pump light first initializes the atomic spins along the *x*-direction, then the pulse of magnetic field creates a transverse spin component, and finally the spins are detected with a pulse of probe light. In this case, the magnetometer signal is a free induction decay 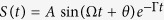, where *θ* is a phase and the amplitude *A* is proportional to the Fourier component of the magnetic field at the Larmor frequency: 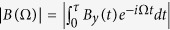, where *τ* is the duration of the magnetic field pulse. For both pulsed and continuous modes, the proportionality constant can be found by applying a known calibration magnetic field.

### Calibration of the pulsed magnetometer

The magnetometer is calibrated by applying a known magnetic field. This calibration field is produced by a coil positioned inside the magnetic shield; the field points in the *y*-direction and has the temporal shape of a single sinusoidal oscillation *B*_cal_(*t*) = *B*_cal_ sin(Ω_cal_*t*) with amplitude *B*_cal_ = 2 nT, frequency Ω_cal_ = 2*π* · 400Hz and Fourier component |*B*_cal_(Ω_cal_)| = *πB*_cal_/Ω_cal_ = 2.5 nT·ms.

In the pulsed mode, the calibration field is applied in between the pump and probe pulses. The recorded magnetometer signal (the free induction decay) is shown in Supplementary Fig. 2(a). The spectrum (calculated as the square-root of the power spectral density 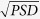) is peaked at the Larmor frequency of the atoms Ω = 2*π* · 400 Hz [Supplementary Fig. 2(b)]. The peak amplitude is proportional to the magnetic field Fourier component: 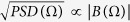. The proportionality constant can be calculated from the data in Supplementary Fig. 2(b) and the known Fourier component of the calibration field. With this calibration we can calculate the Fourier component |*B*_nerve_(Ω)| of the nerve magnetic field [see Fig. 2(f)] from the measured peak values 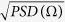 [see Fig. 2(e)].

Supplementary Fig. 2(a,b) also show the magnetometer signal without the applied calibration field. The signal was averaged 1000 times before recorded. The noise at the Larmor frequency is 10200 times smaller than the signal obtained with the calibration field, i.e., the calibration field is detected with a *SNR* = 10200 corresponding to a minimal detectable magnetic field Fourier component 2.5 nT·ms/10200 = 0.25 pT·ms using 1000 averages. The single shot minimal detectable Fourier component is then 7.7 pT·ms.

### Calibration of the continuous magnetometer

In the continuous mode, the lasers are on during the applied calibration field. In this case the magnetometer signal is proportional to the convolution of the magnetic field with the magnetometer response function. Supplementary Fig. 2(c) top shows the detected signal together with a fit to the function 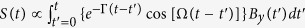 from which we can determine the Larmor frequency and the coherence time. Using the fitted parameters we can perform numerical deconvolution and by scaling with the amplitude of the calibration field we can obtain the magnetic field as a function of time *B*_*y*_(*t*) [Supplementary Fig. 2(c) bottom]. We see that the deconvolution procedure works well as the deconvolved signal resembles a single sinusoidal oscillation.

Supplementary Fig. 2(d) shows the calculated noise spectrum of the deconvoluted signal. It also shows the noise spectrum when the calibration field was off. In this case the noise at the Larmor frequency is 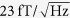 when 100 averages are used. The single shot magnetic field sensitivity is then 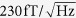.

## Additional Information

**How to cite this article**: Jensen, K. *et al*. Non-invasive detection of animal nerve impulses with an atomic magnetometer operating near quantum limited sensitivity. *Sci. Rep.* **6**, 29638; doi: 10.1038/srep29638 (2016).

## Supplementary Material

Supplementary Information
