# Supplementary material for: Non-invasive detection of animal nerve impulses with an atomic magnetometer operating near quantum limited sensitivity
Source: Sci Rep. 2016 Jul 15;6:29638. doi: 10.1038/srep29638 (PMC4945862; doi:10.1038/srep29638)
Supplement: Supplementary Information [file srep29638-s1.pdf]

Supplementary Information to Non-invasive detection of animal nerve impulses with an atomic magnetometer operating near quantum limited sensitivity.

Kasper Jensen,[1] Rima Budvytyte,[1] Rodrigo A. Thomas,[1] Tian Wang,[1] Annette M. Fuchs,[2] Mikhail V. Balabas,[1,3] Georgios Vasilakis,[1] Lars D. Mosgaard,[1] Hans C. Stærkind,[1] Jörg H. Müller,[1] Thomas Heimburg,[1] Søren-Peter Olesen,[2] and Eugene S. Polzik[1]

[1]Niels Bohr Institute, University of Copenhagen, Blegdamsvej 17, 2100 Copenhagen, Denmark

[2]Department of Biomedical Sciences, Faculty of Health and Medical Sciences, University of Copenhagen, Blegdamsvej 3, 2200 Copenhagen N, Denmark

[3]Department of Physics, St Petersburg State University, Universitetskii pr. 28, 198504 Staryi Peterhof, Russia

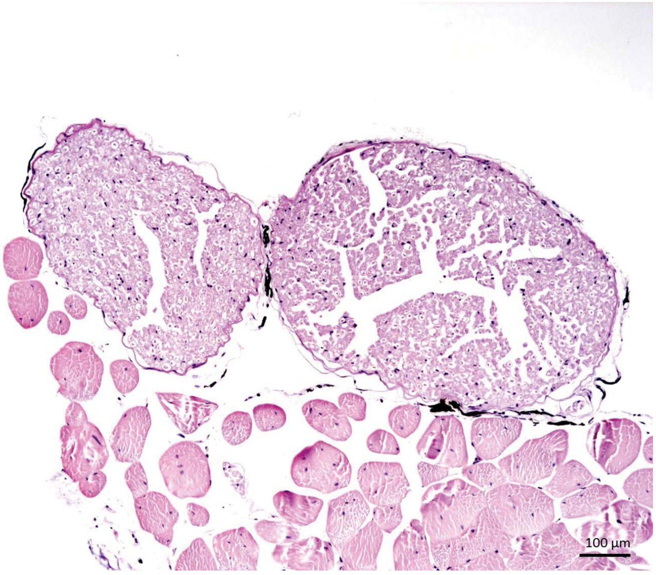

Supplementary Figure 1: Electron micrograph of a frog sciatic nerve. The micrograph shows the cross section of the frog sciatic nerve on lower femur after its first division. The two nerve bundles are surrounded by a fascia of connective tissue, and they are seen above sections of skeletal muscle. The diameters of the two nerve bundles are 0.59 and 0.44 mm and they each contain 1750 and 950 single axons with a minimal diameter of 7 μm. The individual myelinated nerve fibres have an average diameter of 16 μm.

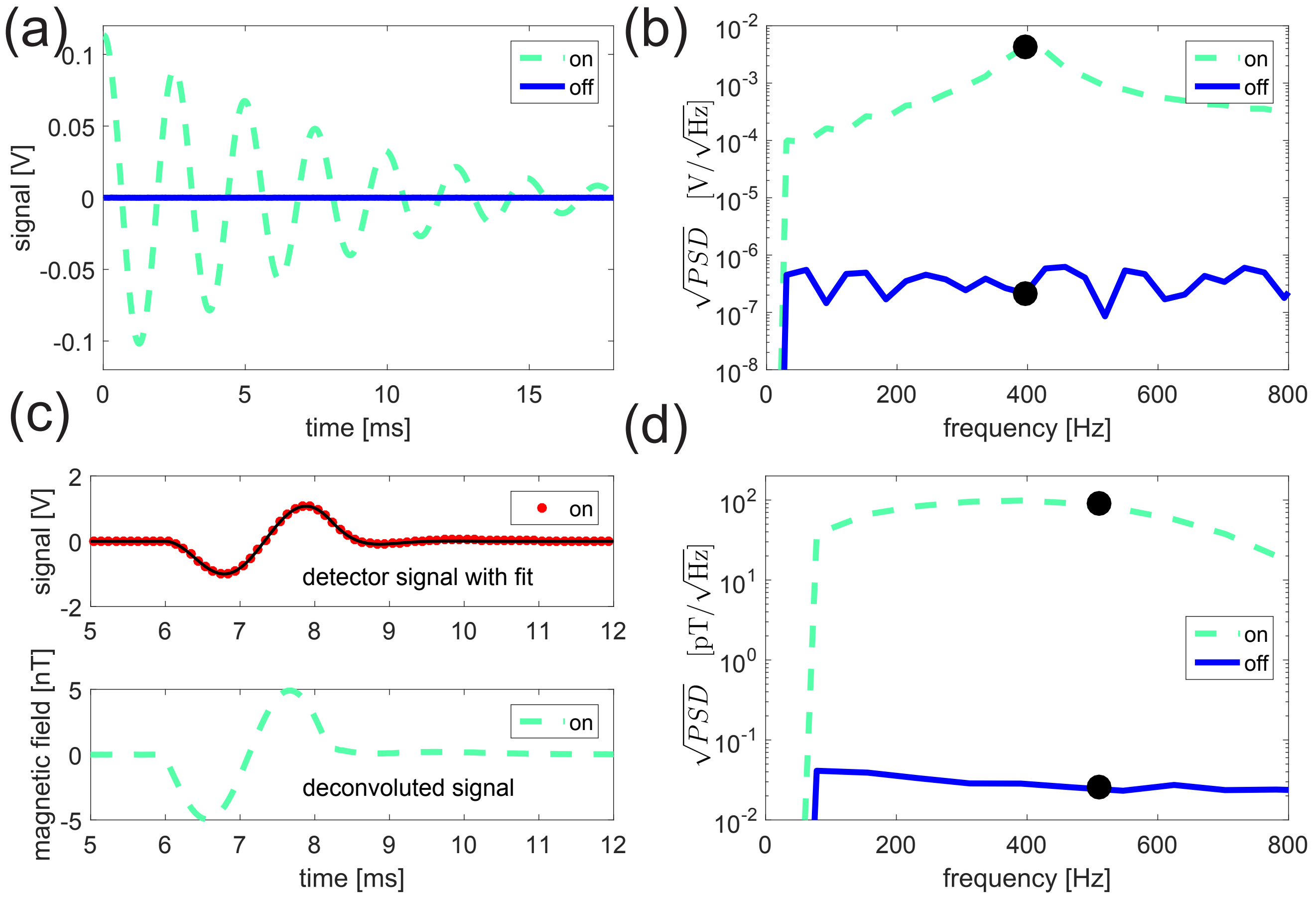

Supplementary Figure 2: Measurements with and without the calibration field. (a,b) Pulsed mode: $\Omega = 400$ Hz, $T_2^{\rm dark} = 27$ ms. The PSD is calculated using the first 18 ms of the recorded signal. (c,d) Continuous mode: $\Omega = 510$ Hz, $T_2 = 0.37$ ms. The PSD is calculated using 7 ms of the recorded signal. The Larmor frequencies are marked in (b) and (d) with a dot.

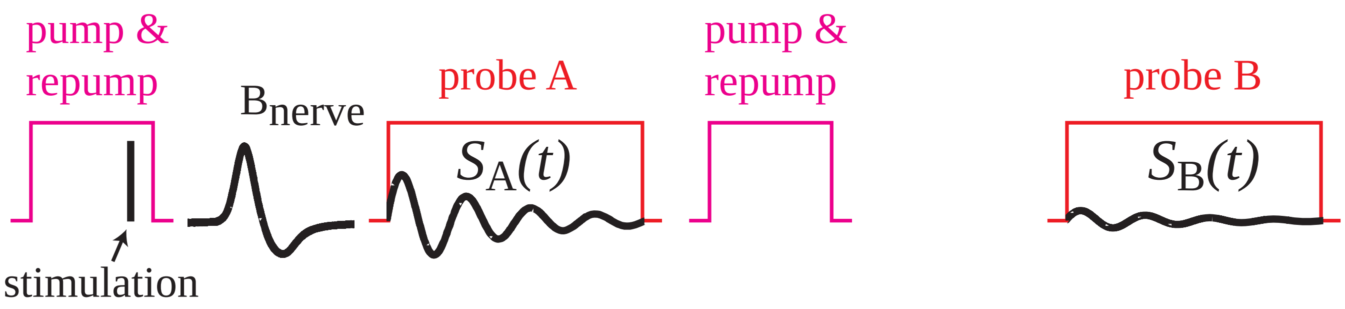

Supplementary Figure 3: Pulse sequence for measuring the nerve impulse.

MAGNETOMETER PRINCIPLE

The total spin of the atomic ensemble is defined as $\mathbf{J} = (J_x, J_y, J_z)$. Here $\mathbf{J}$ is a quantum operator, and the components have the commutation relation $[J_y, J_z] = iJ_x$. $\mathbf{J}$ is here defined as being unitless and equals the total angular momentum divided by the reduced Planck constant $\hbar$. The equations of motion for the spin vector can be derived using the Heisenberg equation of motion

$$\dot{\mathbf{J}}(t) = \frac{1}{i\hbar}\left[\mathbf{J}(t), \mathcal{H}_B\right]. \tag{1}$$

The dot denotes the time-derivative and the bracket denotes the commutator. The Hamiltonian describing the coupling between the spin and the magnetic field is

$$\mathcal{H}_B = \hbar\gamma\mathbf{B}\cdot\mathbf{J}, \tag{2}$$

where γ is the gyromagnetic ratio which for the cesium atom in the $F = 4$ ground state equals 2.2×10^{10} rad/(sec·Tesla). In vector form the equation of motion reads

$$\dot{\mathbf{J}}(t) = \gamma\mathbf{J}\times\mathbf{B}. \tag{3}$$

In the experiment, the atoms are spin-polarized in the x-direction and located in a static magnetic field B_x pointing in the x-direction. In the presence of a small time-dependent magnetic field $B_y(t)$ or $B_z(t)$ pointing in the y- or z-direction, the spin vector will acquire a transverse component $\mathbf{J}_\perp = (J_y, J_z) = |\mathbf{J}_\perp|(\cos\theta, \sin\theta)$. We will assume that J_x is large compared to J_y and J_z, and that J_x is independent of time. We now introduce spin operators J'_y and J'_z rotating at the Larmor frequency $\Omega = \gamma B_x$:

$$\begin{pmatrix} J'_y \\ J'_z \end{pmatrix} = \begin{pmatrix} \cos\Omega t & \sin\Omega t \\ -\sin\Omega t & \cos\Omega t \end{pmatrix}\begin{pmatrix} J_y \\ J_z \end{pmatrix}. \tag{4}$$

In the rotating frame, the equations of motion read

$$\dot{J}'_y(t) \;=\; \gamma J_x\left[\cos(\Omega t)\,B_z(t) - \sin(\Omega t)\,B_y(t)\right] - \Gamma J'_y(t) + \sqrt{2\Gamma}F_y(t), \tag{5}$$

$$\dot{J}'_z(t) = -\gamma J_x\left[\sin(\Omega t)\,B_z(t) + \cos(\Omega t)\,B_y(t)\right] - \Gamma J'_z(t) + \sqrt{2\Gamma}F_z(t). \tag{6}$$

The transverse spin component will eventually decay, and we have therefore added decay terms in the above equations. The decay rate is denoted by Γ and the associated decay time is $T_2 = 1/\Gamma$. We also added Langevin noise operators $F_y(t)$ and $F_z(t)$ with zero mean values and correlation functions $\langle F_y(t)F_y(t')\rangle = \mathrm{var}(F_y)\,\delta(t-t')$, $\langle F_z(t)F_z(t')\rangle = \mathrm{var}(F_z)\,\delta(t-t')$ and $\langle F_y(t)F_z(t')\rangle = 0$, where $\mathrm{var}(F_y) = \mathrm{var}(F_z) = |J_x|/2$ and $\delta(t-t')$ is the Dirac delta-function. These equations can be integrated and the solutions are

$$J'_y(t) = e^{-\Gamma t}J'_y(0) + \sqrt{2\Gamma}\int_{t'=0}^{t} e^{-\Gamma\left(t-t'\right)}F_y(t')dt' + \gamma J_x\int_{t'=0}^{t} e^{-\Gamma\left(t-t'\right)}\left[\cos(\Omega t')\,B_z(t') - \sin(\Omega t')\,B_y(t')\right]dt', \tag{7}$$

$$J'_z(t) = e^{-\Gamma t}J'_z(0) + \sqrt{2\Gamma}\int_{t'=0}^{t} e^{-\Gamma\left(t-t'\right)}F_z(t')dt - \gamma J_x\int_{t'=0}^{t} e^{-\Gamma\left(t-t'\right)}\left[\sin(\Omega t')\,B_z(t') + \cos(\Omega t')\,B_y(t')\right]dt'. \tag{8}$$

From these equations we can calculate the mean values and noise proporties of the transverse spin components as a function of time.

Free precession

Assume that the transverse spin has some mean value at $t = 0$ and that it is then left free to precess. At a later time t the transverse spin component in the rotating frame is

$$\langle \mathbf{J}'_\perp(t)\rangle = \langle \mathbf{J}_\perp(0)\rangle \, e^{-\Gamma t}. \tag{9}$$

We see that the mean value decays in time. In the lab frame the transverse spin will perform a damped oscillation.

Atomic response to a pulse of magnetic field

Consider the case where a magnetic field $B_y(t)$ is applied for a duration τ. We assume that $\tau \ll T_2$ such that any decay of the spin components can be neglegted. If initially the transverse spin component is zero $\langle \mathbf{J}_\perp(0)\rangle = 0$, we find

$$\langle \mathbf{J}'_\perp(\tau)\rangle = \gamma J_x \left(\mathrm{Im}[B_y(\Omega)], -\,\mathrm{Re}[B_y(\Omega)]\right) \tag{10}$$

and

$$\left|\langle \mathbf{J}'_\perp(\tau)\rangle\right| = \gamma J_x \left|B_y(\Omega)\right|. \tag{11}$$

Here we have defined the Fourier component of the magnetic field at the Larmor frequency as

$$B(\Omega) = \int_{t'=0}^{\tau} B(t') e^{-i\Omega t'} dt. \tag{12}$$

Similarly, if the magnetic field is applied along the z-direction instead, the transverse spin component will be

$$\left|\langle \mathbf{J}'_\perp(\tau)\rangle\right| = \gamma J_x \left|B_z(\Omega)\right|. \tag{13}$$

We see that magnetic fields in y- and z-directions have similar effects on the spins: the fields create transverse spin components with lengths proportional to the Fourier components of the magnetic fields at the Larmor frequency.

For the specific case of a sinusoidal magnetic field $B_y(t) = B_0 \sin(\Omega t)$ applied for one period of oscillation $\tau = 2\pi/\Omega$, we find $|B_y(\Omega)| = \pi B_0/\Omega$ and $|\langle \mathbf{J}'_\perp(\tau)\rangle| = \gamma J_x\,(\pi B_0/\Omega)$.

Projection noise limited detection

The measurement of the transverse spin component is fundamentally limited by the spin-projection noise originating from the Heisenberg uncertainty principle. This uncertainty is $\Delta\,|J_\perp| = \sqrt{J_x/2}$. By equating the created mean value given by Eq. (13) to the projection noise we find the uncertainty on the magnetic field Fourier component due to the projection noise:

$$\Delta\left|B_{\mathrm{PN}}(\Omega)\right| = 1/\left(\gamma\sqrt{2J_x}\right). \tag{14}$$

We can also calculate the uncertainty on the amplitude of an oscillating magnetic field due to the projection noise. For a sinusoidal magnetic field with total duration τ equal to an integral multiple of the Larmor period, the amplitude B_0 is related to the Fourier component by $|B(\Omega)| = B_0\tau/2$. From this and Eq. (14) we find the projection noise limited uncertainty on the amplitude:

$$\Delta B_{\mathrm{PN}} = 1/\left(\gamma\sqrt{J_x/2}\tau\right), \tag{15}$$

which is often called the minimal detectable field. The magnetic field sensitivity can be found by multiplying ΔB_{PN} with the square-root of the total measurement time $\sqrt{T_{\mathrm{tot}}}$ and setting $T_{\mathrm{tot}} = \tau = T_2$:

$$\Delta B_{\mathrm{PN}}\sqrt{T_{\mathrm{tot}}} \sim 1/\left(\gamma\sqrt{T_2 J_x/2}\right). \tag{16}$$

The Standard Quantum Limit

Besides the projection noise, the magnetic field measurement will be limited by the quantum shot noise of the probing light, and the back-action noise imposed by the probe on the atomic spins. The total uncertainty is

$$\Delta B_{\mathrm{tot}} = \sqrt{\left(\Delta B_{\mathrm{PN}}\right)^2 + \left(\Delta B_{\mathrm{SN}}\right)^2 + \left(\Delta B_{\mathrm{BAN}}\right)^2}, \tag{17}$$

which can be written as[1,2]

$$\Delta B_{\mathrm{tot}} = \Delta B_{\mathrm{PN}} \cdot \sqrt{1 + 1/\kappa^2 + \kappa^2/3}, \tag{18}$$

where $\kappa = \Delta B_{\mathrm{PN}}/\Delta B_{\mathrm{SN}}$ is a dimensionless light-atom coupling constant. For small coupling strengths, the measurement noise will be dominated by the shot noise, and for large coupling strengths, the measurement noise will be dominated by the back-action noise. By minimizing the uncertainty given by Eq. (18), we find the optimal coupling strength $\kappa \approx 1.3$ and the standard quantum limit on the magnetic field measurement

$$\Delta B_{\mathrm{SQL}} \approx 1.5 \cdot \Delta B_{\mathrm{PN}}, \tag{19}$$

which is ≈ 1.5 times larger than the uncertainty due to the projection noise.

Measuring the atomic signal

The atomic spin can be measured optically. Assume that a linearly polarized pulse of light is propagating in the z-direction through the atomic ensemble. The polarization of the light will be rotated by an angle proportional to J_z due to the Faraday paramagnetic effect. The polarization of the light is described using Stokes operators $S_x(t)$, $S_y(t)$, and $S_z(t)$ which have the unit of 1/time. Here $S_x(t) = \left[\Phi_x(t) - \Phi_y(t)\right]/2$ equals one half the difference in photon flux of x- and y-polarized light. $S_y(t)$ refer to the differences of $+45°$ and $-45°$ polarized light, and $S_z(t)$ to the differences of right hand and left hand circular polarized light. Assuming that the input light before the atomic ensemble is either

x or y-polarized (such that $S_x(t)$ is a large quantity) and that the rotation angle is small, the output light after the atomic ensemble can be described by the equation

$$\begin{aligned} S_y^{\rm out}(t) &= S_y^{\rm in}(t) + aS_x(t)J_z(t) \\ &= S_y^{\rm in}(t) + aS_x(t)\left[\sin(\Omega t)\, J_y'(t) + \cos(\Omega t)\, J_z'(t)\right]. \end{aligned} \tag{20}$$

The parameter a describes the coupling strength between the atoms and the light[1]. The Stokes operator $S_y^{\rm out}(t)$ can be measured with polarization homodyning. There are several ways that one can extract information about the transverse spin components and therefore about the magnetic field from the measured signal. One can for instance measure the mean value $\langle S_y^{\rm out}\rangle$ or the power spectral density of the signal. The power spectral density (PSD) for a function $x(t)$ is defined as

$$S_{xx}(\omega) = \frac{1}{T}\left\langle \left| \int_{t=0}^{T} x(t)e^{-i\omega t}dt \right|^2 \right\rangle = \frac{1}{T}\int_{t=0}^{T}\int_{t=0}^{T} \langle x(t)x(t')\rangle\, e^{-i\omega(t-t')}dtdt'. \tag{21}$$

We will show below that the PSD of $\langle S_y^{\rm out}(t)\rangle$ is proportional to the amplitude squared of the applied magnetic field.

Detection of a pulse of magnetic field

Assume that a pulse of magnetic field $B_y(t)$ of duration τ is applied from $t = -\tau$ to $t = 0$. After the pulse, the spins have acquired a non-zero transverse spin component $\langle \mathbf{J}'_\perp(0)\rangle \propto |B_y(\Omega)|$ as given by Eq. (11). At $t = 0$ the spin will continue to precess until it decays as described by Eq. (9). This spin vector can be measured using a pulse of light with duration T and starting at the time when the magnetic field pulse ends. The mean value of the measured signal is

$$\langle S_y^{\rm out}(t)\rangle = aS_x(t)\left[\sin(\Omega t)\langle J_y'(0)\rangle + \cos(\Omega t)\langle J_z'(0)\rangle\right]e^{-\Gamma t} = aS_x(t)\,|\langle \mathbf{J}'_\perp(0)\rangle|\sin(\Omega t+\theta)\, e^{-\Gamma t}, \tag{22}$$

where θ is the polar angle of $\langle \mathbf{J}'_\perp(0)\rangle$. The amplitude of the transverse spin vector can be extracted from the measurement by, for instance, a fit of the experimental data to Eq. (22). Alternatively, one can calculate the PSD of the signal. For $x(t) = A\sin(\Omega t+\theta)\, e^{-\Gamma t}$ we calculate that the peak value of the PSD is

$$S_{xx}(\Omega) = |A|^2\left[\frac{\left(1-e^{-\Gamma T}\right)^2}{4\Gamma^2} + \epsilon(\theta,\Gamma,\Omega,T)\right], \tag{23}$$

where the second term $\epsilon(\theta,\Gamma,\Omega,T)$ is much smaller than the first term for our experimental parameters. We see that

$$S_{xx}(\Omega) \propto |\langle \mathbf{J}'_\perp(0)\rangle|^2 \propto |B_y(\Omega)|^2, \tag{24}$$

and that the Fourier component of the magnetic field at the Larmor frequency can be extracted from the peak value of the PSD.

Continuous recording of the magnetic field

We will now discuss how one can measure the magnetic field as a function of time. Assume that a magnetic field $B_y(t)$ is applied and that light is continuously monitoring the atomic spin. If $\langle \mathbf{J}_\perp(0)\rangle = 0$, then at a later time

$$\langle J_z(t)\rangle = -\gamma J_x \int_{t'=0}^{t} e^{-\Gamma(t-t')} \cos\left[\Omega\left(t-t'\right)\right] B_y(t')dt'. \tag{25}$$

The mean value of the measured signal will be

$$\left\langle S_y^{\mathrm{out}}(t)\right\rangle = aS_x(t)\left\langle J_z(t)\right\rangle. \tag{26}$$

From this we see that the measured signal $\left\langle S_y^{\mathrm{out}}(t)\right\rangle$ is proportional to the convolution of $B_y(t)$ with the function $\left[-\cos\left(\Omega t\right)e^{-\Gamma t}\right]$. Similarly, if the transverse magnetic field is pointing in the z-direction, the signal is proportional to the convolution of the magnetic field $B_z(t)$ with the function $\left[\sin\left(\Omega t\right)e^{-\Gamma t}\right]$. The magnetic field as a function of time can be extracted from the measured data using numerical deconvolution.

EXPERIMENTAL PROCEDURE

Three lasers denoted pump, repump and probe are used in the experiment. The pump laser is on resonance with the cesium $F = 4 \to F' = 4$ D1 transition and has the wavelength 895 nm. The repump laser is on resonance with the cesium $F = 3 \to F' = 2, 3, 4$ D2 transitions (all are within the Doppler linewidth) and has the wavelength 852 nm. These two lasers are used for optical pumping of the cesium atoms into the $F = 4, m = 4$ hyperfine sublevel and are thereby creating a high spin-polarization of the cesium vapor. The probe laser is 1.6 GHz higher in frequency than the cesium $F = 4 \to F' = 5$ D2 transition and has the wavelength 852 nm. The 1.6 GHz detuning is much larger than both the natural linewidth (5 MHz FWHM) and the Doppler linewidth (380 MHz FWHM) such that negligible absorption occurs.

The pulse sequence in Supplementary Fig. 3 is used for detection of the magnetic field from the nerve impulse B_{nerve}. The atoms are first optically pumped using pump and repump light, then the magnetic field is present, and finally the atoms are measured using probe pulse A. The optically detected signal $S_A(t)$ will be a free induction decay as seen in Supplementary Fig. 3. Due to misalignment of the pump and repump laser beams with respect to the bias field B_x (see Fig. 1 in the main text), one may observe a free induction decay $S_B(t)$ [see probe pulse B in Supplementary Fig. 3] even if there is no magnetic field. The pump/repump and probe pulses are therefore repeated and the signals from probe pulses A and B are subtracted giving the magnetometer signal $S(t) = S_A(t) - S_B(t)$. The amplitude of this magnetometer signal will be proportional to the Fourier component of the magnetic field at the Larmor frequency $|B(\Omega)|$.

CONDUCTION VELOCITY

The nerve conduction velocity can be calculated by dividing the distance from the stimulation electrodes to the recording site [34(5) mm for optical recording and 53(10) mm for electrical recording] by the time interval between the stimulus artifact and the zero-crossing of the observed nerve signal (see Fig. 4(c) in the main text). The electrical recording measures the potential difference ΔV between two external electrodes separated by 5 mm. As the extent of the action potential Φ is much larger than electrode spacing, the electrical signal is proportional to the time-derivative of the action potential $\Delta V \propto \partial\Phi/\partial t$. The zero-crossing of the electrical signal therefore corresponds to when the peak of the action potential passes the electrodes. Also, when the peak of the action potential passes the magnetometer, the magnetic field is zero, as the magnetic field $B(t) \propto \partial\Phi/\partial t$ according to a simple model for the nerve[3]. The earlier arrival of the nerve impulse for optical recording compared to electrical recording [1.0(2) ms compared to 2.4(2) ms] is consistent with the magnetometer being positioned in between the stimulating and recording electrodes. From the measurements shown in Fig. 4(c) we calculate the conduction velocity of 34(8) m/s and 22(5) m/s for optical and electrical recording. We note that stating a single number for the conduction velocity is not entirely correct as the frog sciatic nerve contains thousands of axons of varying diameter (see Fig. S1). For a single myelinated axon, the conduction velocity is proportional to the axon's diameter, which leads to a distribution of velocities within the range 10-40 m/s for axons in the frog sciatic nerve[4].

ESTIMATE OF THE AXIAL IONIC CURRENT

The detected magnetic field is created by axial ionic currents inside the nerve bundle. There is a forward current inside the axons and a return current outside the axons. The magnetic fields from the forward and return currents can cancel each other, the exact degree of cancellation depends on the anatomy of the nerve, and the geometry of the experiment, such as the size of the magnetic field sensor and the distance from the nerve to the sensor.

We can estimate the axial current in the nerve from our magnetic field measurements. We use a simple model, where we assume that the ionic current is concentrated at the center of the nerve, and that the nerve produces a magnetic field similar to that of an infinitely long conducting wire. The magnetic field from an infinitely long wire is $|B| = \mu_0 I/(2\pi r)$, where μ_0 is the magnetic permeability, I is the current, and r is the radial distance from the wire. Using $r = 1.9$ mm for the distance from the center of the nerve to the center of the vapor cell, we calculate that a current of 0.23 μA will produce a magnetic field of 24 pT.

Our estimate of 0.23 μA is smaller than the 0.4 μA which was estimated in previous work on the frog sciatic nerve[5]. This is expected as in that work, the nerve was put in a large container with saline solution and the magnetic field was measured by a coil with the nerve inside it, such that a large part of the return current could flow without being detected.

[1] K. Jensen. Quantum information, entanglement and magnetometry with macroscopic gas samples and non-classical light. PhD thesis, University of Copenhagen (2011).

[2] Vasilakis, G. et al. Generation of a squeezed state of an oscillator by stroboscopic back-action-evading measurement. *Nat. Phys.*, **11,** 389–392 (2015).

[3] Barry, J. F. et al. Optical magnetic detection of single-neuron action potentials using quantum defects in diamond. arXiv:1602.01056 (2016).

[4] Wijesinghe, R. S., Gielen, F. L. H. & Wikswo, J. P. A model for compound action potentials and currents in a nerve bundle III: A comparison of the conduction velocity distributions calculated from compound action currents and potentials. *Ann. Biomed. Eng.*, **19,** 97–121 (1991).

[5] Wijesinghe, R. S., Gielen, F. L. H., & Wikswo, J. P. A model for compound action potentials and currents in a nerve bundle I: The forward calculation. *Ann. Biomed. Eng.*, **19,** 43–72 (1991).
